# Clinical implications of central blood pressure measured by radial tonometry and automated office blood pressure measured using automatic devices in cardiovascular diseases

**DOI:** 10.3389/fcvm.2022.906021

**Published:** 2022-08-11

**Authors:** Ami Kwon, Gee-Hee Kim, Min-Sik Kim

**Affiliations:** ^1^Division of Cardiology, Department of Internal Medicine, Seoul St. Mary’s Hospital, College of Medicine, The Catholic University of Korea, Seoul, South Korea; ^2^Division of Cardiology, Department of Internal Medicine, St. Vincent’s Hospital, College of Medicine, The Catholic University of Korea, Seoul, South Korea; ^3^Catholic Research Institute for Intractable Cardiovascular Disease, College of Medicine, The Catholic University of Korea, Seoul, South Korea

**Keywords:** blood flow velocity, carotid artery, arterial stiffness, atherosclerotic cardiovascular disease, central blood pressure

## Abstract

**Objective:**

Central aortic systolic blood pressure (CBP) measured by carotid-femoral pulse wave analysis (cfPWA) is a gold standard method to estimate true arterial pressure. However, the impact of the CBP level measured by radial PWA on cardiovascular (CV) risk assessment is unclear. This study aimed to determine the impact on CV outcome assessment and the association between the optimal levels of non-invasively measured CBP and automated office blood pressure (OBP) in clinical practice.

**Materials and methods:**

A total of 2,115 patients underwent non-invasive semiautomated radial artery applanation tonometry (Omron HEM-9000AI) in the Department of Internal Medicine, St. Vincent’s Hospital, from July 2011 to December 2015. The patients were followed for at least 5 years, and atherosclerotic cardiovascular (ASCVD) outcomes were collected.

**Results:**

Among 2,115 patients (mean age 58 ± 14 years, 50.4% men) who were followed up, the median follow-up period was 52 months (range: 1–104 months). The total number of patients with ASCVD events was 163 (7.70%). In multivariate Cox regression analysis, a CBP of more than 125 mmHg and an automated OBP of more than 131 mmHg were independently associated with a significant increase in ASCVD outcomes. After adjusting for confounding factors, the hazard ratio for ASCVD events increased by 12.5, 11.7, and 12.7%, for every 10 mmHg increase in automated OBP, CBP, and central pulse pressure (PP), respectively.

**Conclusion:**

This study demonstrated that the automated OBP measured using the method used in real clinical practice and CBP measured by radial tonometry were associated with an increased risk for adverse ASCVD outcomes.

## Introduction

Increased blood pressure (BP), usually measured *via* the brachial artery, is a major risk factor for cardiovascular disease (CVD), and lowering BP might improve the prognosis of CVD and prevent CVD events and death (1). Among several different BP measurement techniques, the auscultatory technique involving a trained observer and mercury sphygmomanometer continues to be a gold standard method of choice for manual measurement in the office (2). However, in the mercury-free era, automated office systolic BP (SBP) measurements using automatic devices have helped to improve and simplify the technical aspects of the measurement but need to be further studied (3, 4). In addition, automated office SBP readings are lower than manual readings in the clinic or office potentially due to a reduction in the white coat effect (5). Additionally, automated measurements are a relatively easy way to take multiple measurements in a short period of time without adversely affecting accuracy (6). However, further studies regarding the impact of automated office SBP on cardiovascular (CV) risk assessment are needed.

Arterial pressure varies continuously throughout the cardiac cycle, and the shape of the pressure waveform changes continuously throughout the arterial tree. Although diastolic and mean arterial pressures are relatively constant, systolic pressure may be up to 40 mmHg higher in the brachial artery than in the aorta (7). This phenomenon of systolic pressure amplification arises principally because of an increase in arterial stiffness as the distance from the heart increases. In addition, arterial stiffness can exacerbate changes in arteries due to the aging process or accelerated cardiovascular risk or inflammation (8). Therefore, brachial pressure is a poor surrogate for aortic pressure (9). Several lines of evidence suggest that non-invasively-determined central pressure including pulse pressure (PP) is also more strongly related to vascular hypertrophy, the extent of atherosclerosis, and CV events than brachial BP (10, 11). Central aortic systolic blood pressure (CBP) measured by carotid-femoral pulse wave analysis (cfPWA) is a novel method to estimate true arterial pressure. However, carotid waveforms of sufficient quality can be difficult to obtain in all individuals, especially in obese patients (12). Estimating the CBP in the radial artery by measuring not only the ordinary brachial BP but also the radial arterial pulse wave may enable a more accurate evaluation of changes in central aortic pressure during vasodilator therapy, and this method is also relatively easy but does not adversely affect accuracy (13). Further investigations of CBP measured in the radial artery are needed. Additionally, the impact of the CBP level measured by radial PWA on CV risk assessment and the relationship between CBP and automated office SBP are unclear. Therefore, this study aimed to determine the impact of CBP measured by radial PWA and assess CV risk assessment and the association between non-invasively measured optimal values of CBP and SBP in clinical practice.

## Materials and methods

### Study population

Among patients who underwent non-invasive, semiautomated, radial artery applanation tonometry (Omron HEM-9010AI) in the Department of Internal Medicine at St. Vincent’s Hospital from July 2011 to December 2015, a total of 2,115 subjects (1,066 men; mean age: 58 ± 14 years) were enrolled in this study. Subjects who had an irregular cardiac rhythm were excluded due to the method used to measure radial PWA.

There was no industry involvement in the design, implementation, or data analysis of this study. The present study was a single-center retrospective study and was approved by the Institutional Review Board (VC19RISI0266).

### Measurement of brachial blood pressure and central blood pressure

The participants rested for at least 5 min in a quiet room prior to the BP measurement. The patients were comfortably seated with their legs uncrossed and their back and arms supported. Brachial BP was measured using an HEM-907 automatic cuff oscillometric device (Omron Healthcare). The average of three readings was obtained for automated office SBP, diastolic BP (DBP), PP, and heart rate (HR). Next, the radial PWA was examined with a HEM-9010AI automated applanation tonometer (Omron Healthcare) in the same way as our previous research (14).

### Clinical and biochemical assessments

Blood specimens were obtained after a 12 to 14-h fast (8:00 p.m.–9:30 a.m.) to reduce the influence of circadian variation. Total cholesterol (TC) and triglyceride (TG) concentrations were assessed by using standard enzyme methods. The high-density lipoprotein (HDL) cholesterol level was measured after precipitation of very-low-density lipoproteins and low-density lipoprotein (LDL) with phosphotungstic acid, and LDL was calculated using the Friedewald formula. Fasting glucose levels were enzymatically measured by the hexokinase method. A blood sample from every patient was drawn and centrifuged within 30 min. The serum samples were stored at −80°C, and high sensitivity C-reactive protein (hs-CRP) was determined using an immunoturbidity assay (Liatest; Stago, Asnieres-sur-Seine, France), with an interassay variability coefficient of variation of 6.25%. The above method proceeded in the same way as in our previous study (15).

### Transthoracic Doppler echocardiography

Two-dimensional, M-mode, pulsed Doppler, and tissue Doppler echocardiography were performed using a Vivid 7 ultrasound machine (GE Medical Systems, Horten, Norway) with a 2.5-MHz transducer. Standard two-dimensional measurements (left ventricle diastolic and systolic dimensions, ventricular septum and posterior wall thickness, and left atrial volume) were assessed as recommended by the American Society of Echocardiography (16). The mitral inflow velocities were traced and the following variables were obtained: peak velocity of early diastolic mitral inflow, late diastolic mitral inflow, and deceleration time of the E velocity. Early diastolic mitral annular, late diastolic, and systolic velocities of the mitral annulus were measured from the apical four-chamber view at the septal corner of the mitral annulus. The above method proceeded in the same way as in our previous study (15).

### Outcomes

The primary endpoints were defined as the cumulative incidence of atherosclerotic CVD (ASCVD) events or death or high blood pressure (HBP) complications (such as LV hypertrophy, retinopathy, and proteinuria), or heart failure. Medical records were obtained from ASCVD-related physician visits during follow-up and were reviewed by cardiologists. ASCVD was defined as the presence of acute coronary syndrome [ACS, including ST-elevation myocardial infarction (MI), non-ST elevation MI, and unstable angina] or a history of MI, stable or unstable angina, coronary or other arterial revascularization, cerebrovascular diseases (CVA), including stroke or transient ischemic attack, or peripheral arterial disease (PAD), defined as an ankle-brachial index < 0.9 measured, using an Omron VP-1000 Vascular Profiler (Omron Healthcare, Kyoto, Japan) presumed to be of an atherosclerotic origin.

### Statistical analyses

Continuous variables are presented as mean ± standard deviation (SD), and categorical variables are presented as absolute and relative frequencies (%). A *t*-test was used to compare the means when there were two groups. Proportions were compared using the two-way tables and chi-square tests. Multivariate analyses using the Cox proportional hazard regression model were applied to the variables that were significant in the univariate analysis and known important risk factors for ASCVD. In addition, multivariate analyses were schematized using a restricted cubic spline curve. Two-sided *p*-values of ≤ 0.05 indicated statistical significance. Statistical analyses were conducted using SAS 9.1 statistical software (SAS Institute, Cary, NC, United States) and R version 3.6.3 (The R Foundation for Statistical Computing, Vienna, Austria^1^).

## Results

### Patient characteristics

The characteristics of the study population and the mean values of the individual parameters of the arterial pressure are detailed in Table 1. A total of 2,115 patients were included in this study, and the median follow-up time was 52 months (range: 1–104 months). The mean age at the time of examination was 58 ± 14 years, 50.4% were men, and the average body mass index (BMI) was 24.5 ± 3.4 kg/m^2^. A history of HBP was present in 29.0% of the population, 27.0% of subjects were taking renin-angiotensin inhibitors, and 14.2% were taking beta-blockers. A total of 29.1% of enrolled patients were taking statins. Diabetes mellitus (DM) was present in 6.5% of participants. There was no difference between the groups in regard to BMI or a history of HBP. Patients with ASCVD events were older, were more frequently male, and were more likely to have a history of DM, smoking, coronary artery disease (CAD), and CVA than those who did not have ASCVD events. Baseline SBP, CBP, and HR were similar in both groups. However, PP was higher in the ASCVD event group. Lipid profiles were similar in both groups, and serum creatinine levels were higher in the ASCVD event group. The percentage of patients taking antihypertensive medications and lipid-lowering medications was significantly higher in the ASCVD event group.

**TABLE 1:** Baseline characteristics and comparison of demographic variables and cardiovascular risk factors according to incident primary endpoints during follow up period.

	Total	No	Yes	*P*-value
Number (%)	2,115	1,850 (87.5)	265 (12.5)	
Male (%)	1,066 (50.4)	909 (49.1)	157 (59.2)	0.003
Age [mean (SD)]	57.9 (13.6)	57.1 (13.8)	63.9 (10.4)	<0.001
BMI [mean (SD)]	24.5 (3.4)	24.45 (3.4)	24.61 (3.4)	0.472
HBP (%)	613 (29.0)	565 (30.5)	48 (18.1)	<0.001
DM_(%)	138 (6.5)	123 (6.6)	15 (5.7)	0.634
Smoking (%)	577 (27.3)	487 (26.3)	90 (34.0)	0.016
CAD (%)	1,927 (91.1)	1,683 (91.0)	244 (92.1)	0.635
CVA (%)	13 (0.6)	9 (0.5)	4 (1.5)	0.116
Automated OBP [mean (SD)]	131 (18.8)	131 (18.5)	135 (20.8)	0.006
PP [mean (SD)]	54 (14.3)	53 (13.8)	59 (16.5)	<0.001
CBP [mean (SD)]	121 (19.8)	121 (19.6)	124 (21.2)	0.02
DBP [mean (SD)]	78 (12.5)	78 (12.5)	76 (12.4)	0.015
Central PP [mean (SD)]	44 (15.1)	43 (14.7)	48 (16.8)	<0.001
HR [mean (SD)]	72 (12.5)	73 (12.6)	71 (12.0)	0.178
Radial AIx [mean (SD)]	80.2 (13.5)	80.1 (13.6)	81.2 (12.4)	0.182
TC [mean (SD)]	186 (42.2)	187 (41.8)	180 (43.8)	0.01
LDL [mean (SD)]	111 (35.8)	112 (35.8)	106 (35.0)	0.009
HDL [mean (SD)]	44 (11.6)	44 (11.6)	41 (10.7)	<0.001
Cr [mean (SD)]	0.87 (0.50)	0.86 (0.51)	0.93 (0.36)	0.023
ACEiARB (%)	571 (27.0)	470 (25.4)	101 (38.1)	<0.001
BB (%)	301 (14.2)	233 (12.6)	68 (25.7)	<0.001
Statin (%)	616 (29.1)	500 (27.0)	116 (43.8)	<0.001

Data are mean ± SD or percentage as marked. *P*-value: independent *t*-test analysis of variance for numeric variables and chi-square test for categoric variables.

OBP, office blood pressure; PP, pulse pressure; CBP, central blood pressure; Aix, augmentation index; TC, total cholesterol; LDL, low-density lipoprotein; HDL, high-density lipoprotein; Cr, creatinin; RASi, renin-angiotensin system inhibitor; ARB, renin-angiotensin receptor blocker; BB, beta-blocker.

### Follow-up and independent predictors of cardiovascular disease

Among the entire cohort, the total number of patients with primary endpoints was 265 (12.5%). In total, 42 patients (1.98%) died, seven were cardiac deaths and 35 were deaths due to other causes. ACS events developed in 106 patients, including 13 patients with MI events. Additionally, 33 patients had CVA events, and 14 patients suffered from PADs. Furthermore, 21 patients had heart failure, of which the most common etiology was dilated cardiomyopathy. HBP complications developed in 49 patients.

The results of the univariate Cox proportional hazard regression analysis of the relationships of each BP value with ASCVD outcomes are shown in Table 2. According to the Cox regression model, automated office SBP (*p* = 0.046), CBP (*p* = 0.004), and central PP (*p* < 0.001) were significant determinants of ASCVD events. Additionally, the multivariate analysis results are presented in Table 3. All relative hazard ratios (*HR*s) were adjusted for age, sex, BMI, CAD, HBP, DM, fasting glucose, and total cholesterol/high-density lipoprotein. After adjusting for confounding factors, the hazard ratio for ASCVD events increased by 12.5, 11.7, and 12.7% for every 10 mmHg increase in SBP, CBP, and central PP, respectively.

**TABLE 2:** Univariable Cox models of relation of each blood pressure variables to primary endpoints.

	Hazard ratios	Confidence interval	*P*-value
Automated OBP (per 10 mmHg)	1.084	1.001–1.173	0.046
CBP (per 10 mmHg)	1.061	0.983–1.145	0.004
Central PP (per 10 mmHg)	1.196	1.092–1.311	<0.001
Radial Alx (per 10 mmHg)	1.030	0.887–1.115	0.619

All blood pressure per 10 mmHg. OBP, office blood pressure; CBP, central blood pressure; PP, pulse pressure; Aix, augmentation index.

**TABLE 3:** Multivariable Cox models of relation of each blood pressure variables to primary endpoints.

	Hazard ratios	Confidence interval	*P*-value
Automated OBP (per 10 mmHg)	1.125	1.038–1.220	0.004
CBP (per 10 mmHg)	1.117	1.034–1.207	0.005
Central PP (per 10 mmHg)	1.127	1.014–1.253	0.027
Radial Alx (per 10 mmHg)	1.068	0.933–1.223	0.34

All blood pressure per 10 mmHg.

OBP, office blood pressure; CBP, central blood pressure; PP, pulse pressure; Aix, augmentation index.

### Derivation of diagnostic thresholds for central aortic systolic blood pressure

**FIGURE 1:**
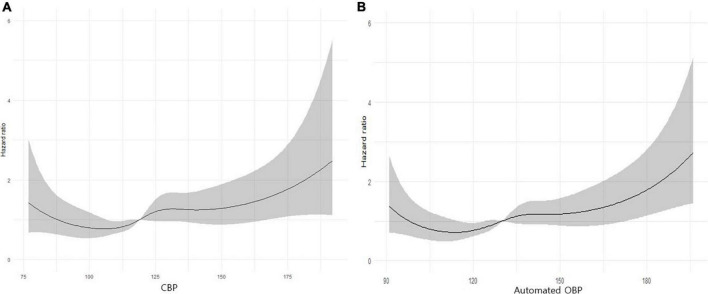
**(A)** A restricted cubic spline curve of cumulative incidence of the atherosclerotic cardiovascular (ASCVD) outcome according to central aortic systolic blood pressure (CBP) values. Statistical analyses were conducted using R ver. 3.6.3 (The R Foundation for Statistical Computing, Vienna, Austria; https://www.r-project.org/). **(B)** A restricted cubic spline curve of cumulative incidence of the ASCVD outcome according to automated office BP (OBP) values. Statistical analyses were conducted using R ver. 3.6.3 (The R Foundation for Statistical Computing, Vienna, Austria; https://www.r-project.org/).

As shown in Figures 1A,B, the restricted cubic spline curve of cumulative incidence of the ASCVD outcome according to SBP and CBP values was analyzed based on the Cox proportional risk model. Through this, the result-driven diagnostic threshold for radial CBP is presented as 120 mmHg for CBP and 131 mmHg for SBP for ASCVD events.

## Discussion

The aim of this study was that suggesting a radial CBP value for predicting ASCVD events, which was compared with automated office SBP for ASCVD prediction. We suggested that measurements greater than 131 mmHg for office BP (OBP) and greater than 120 mmHg for radial CBP may be highly associated with ASCVD event occurrence. Additionally, the risk of ASCVD events increased by 12.5 and 11.7%, with a 95% confidence interval (*CI*), for every 10 mmHg increase in SBP and radial CBP, respectively. Our study was performed in a relatively young age group (mean age 57.9 ± 13.6 years) that was randomly recruited, and the sample was not limited to high–risk patients with CVD, unlike many other SBP and CBP studies that targeted the elderly age group or high-risk CVD groups.

High BP is now the leading cause of cardiovascular mortality and morbidity (17, 18). Therefore, accurately measuring and managing BP is an effective way to prevent ASCVD. In addition, LV hypertrophy is an important end-organ damage consequence of high BP. LVH has been linked with cardiovascular events. To date, the gold standard for BP measurement and the basis of the overwhelming majority of clinical trial databases is a mercury manometer method (2, 19). However, from 2020, the use of mercury sphygmomanometers is difficult, because of the prohibition resulting from the Minamata treaty. Therefore, a BP measurement method that does not use a mercury sphygmomanometer is required. SBP can be measured easily and repeatedly and reduces the white coat effect that impacts manual office BP (OBP). According to some studies, automated OBP is 5–10 mmHg or up to 10–20 mmHg lower than conventional OBP (20, 21). A similar trend was found in our study, the average manual office BP measurement was higher than automated office BP and CBP measurements.

As the first observational and prospective study conducted on this topic, the SPRINT trial is the landmark study regarding intensive BP lowering using automated OBP. The trial was stopped early because the intensive treatment group (target SBP < 120 mmHg) had 25% lower rates of CVD events and a 27% lower risk of death from all causes than the standard treatment group (target SBP < 140 mmHg) (22). Based on this study, 11 hypertension-related societies, such as the American Heart Association and the American Heart Disease Association, have changed the target BP from 140/90 to 130/80 mmHg (23, 24). This trial used only data obtained from an automated device to prescribe and adjust medications during the study. The Action to Control Cardiovascular Risk in Diabetes study, which was another large-scale study that was a randomized controlled trial in which either intensive or standard BP control was implemented, used the same measurement device as that used in SPRINT (25). In our study, an automated OBP greater than 131 mmHg was highly associated with ASCVD event occurrence.

Additionally, we compared SBP and radial CBP in our study. In general, SBP is measured by brachial artery pressure. Brachial SBP may not reliably estimate the real effects of HBP in organs. CBP is a more direct measurement of the pressure to which most major target organs are exposed, and therefore may have the potential to enhance diagnostic and therapeutic value compared with the value associated with conventional BP measurement using a mercury sphygmomanometer. Several studies have examined the longitudinal relationship between central hemodynamic parameters and clinical outcomes (10, 11, 26–29). The Strong Heart Study demonstrated that aortic SBP and PP are independently associated with CV mortality and events and that aortic PP is superior to brachial PP in predicting outcomes (10). Similarly, an Italian study in older patients showed that carotid CBP and PP were independently associated with CV events and that carotid CBP was associated with fatal CV events; the association remained after adjusting for brachial BP (11). According to a recently published study, similar to this study, central PP had a stronger correlation with target organ damage (TOD) than other CBP measurements (30). In the Conduit Artery Functional Evaluation study, the impact of two different BP-lowering regimens (atenolol-based vs. amlodipine-based therapy) was similar in regard to brachial artery pressure, but substantial reductions in central aortic pressures were observed with amlodipine-based therapy. Based on these results, CBP was considered an important surrogate marker of CVD mortality and morbidity in both regimen groups, and measuring CBP rather than brachial BP is more useful for predicting ASCVD outcomes (26). When measuring CBP, direct measurement of the aorta is accurate, but it is invasive and difficult to measure in general clinical practice. Non-invasive applanation tonometry of the radial artery and carotid artery are frequently used (31). Carotid artery applanation tonometry is widely used in the estimation of local carotid artery pressure waveforms and cfPWA. However, it is difficult to assess adequate sites for applanation because the artery should move freely with the sensor, and respiratory artifacts are common. Furthermore, the method could activate baroreceptors, leading to reflexive changes in *HR* and arterial pressure (32, 33). Therefore, the radial artery has been proposed as the best site for non-invasive assessment because optimal flattening force or applanation may be easier to achieve at the radial artery than at other sites (34). The most common non-invasive method to estimate CBP to date has been radial applanation tonometry (31).

Theoretically, radial artery pressure should be higher than brachial artery pressure (34). However, our study showed that the diagnostic value for predicting ASCVD events was lower for radial CBP (greater than 125 mmHg) than for brachial SBP (greater than 131 mmHg). This could be explained by differences in methodologic principles between the conventional brachial oscillometric method and the PWA method. Considering this, follow-up studies comparing the actual diagnostic values of SBP and CBP are needed. In the 24-h ambulatory blood pressure (ABP) study, 24-h central ABP, measured with regular brachial cuffs and dedicated software, tends to be superior to 24-h brachial ASBP in predicting organ damage related to the heart, which is LV hypertrophy (35). Additionally, these researchers suggest upper normal limits for 24-h central SBP of 135 mmHg and for 24-h central SBP of 120 mmHg as measured by invasive and non-invasive gold standard methods (36).

Our study has several limitations. First, automated office SBP was measured by an HEM 9000-IC, which has been validated for home BP monitoring. The device has not been validated for office BP, and we assumed that the accuracy of this fully automated device was validated. Second, radial CBP is not the gold standard method, but our method is an easy and simple tool that can be used in real practice. Third, our study used an observational prospective design. The BP-lowering effect of antihypertensive medications cannot be reasonably interpreted among the enrolled patients based on ASCVD events.

In conclusion, this study demonstrated that the automated office SBP greater than 131 mmHg measured using the method used in clinical practice and CBP greater than 125 mmHg measured by radial tonometry were associated with an increased risk of adverse ASCVD outcomes. This finding suggests that useful and easy non-invasive radial tonometry methods for CBP may be considered. However, further study is needed to determine changes in the occurrence of ASCVD events as a result of targeting brachial SBP and racial CBP.

## Data availability statement

The raw data supporting the conclusions of this article will be made available by the authors, without undue reservation.

## Ethics statement

The studies involving human participants were reviewed and approved by Institutional Review Board (VC19RISI0266). Written informed consent for participation was not required for this study in accordance with the national legislation and the institutional requirements.

## Author contributions

AK was responsible for conceptualization, methodology, software, investigation, data curation, and writing–original draft. G-HK was responsible for conceptualization, formal analysis, investigation, resources, editing of the manuscript, visualization, and project administration. M-SK was responsible for conceptualization, formal analysis, investigation, editing of the manuscript, and visualization. All authors contributed to the article and approved the submitted version.

## Footnotes

1
https://www.r-project.org/

## References

[1] Wang JG Staessen JA Franklin SS Fagard R Gueyffier F. Systolic and diastolic blood pressure lowering as determinants of cardiovascular outcome. *Hypertension.* (2005) 45:907–13. 10.1161/01.HYP.0000165020.14745.79 15837826

[2] Pickering TG Hall JE Appel LJ Falkner BE Graves J Hill MN Recommendations for blood pressure measurement in humans and experimental animals: part 1: blood pressure measurement in humans: a statement for professionals from the Subcommittee of Professional and Public Education of the American Heart Association Council on High Blood Pressure Research. *Hypertension.* (2005) 45:142–61. 10.1161/01.HYP.0000150859.47929.8e 15611362

[3] Drawz P. Clinical implications of different blood pressure measurement techniques. *Curr Hypertens Rep.* (2017) 19:54. 10.1007/s11906-017-0751-0 28551831

[4] Vischer AS Burkard T. Principles of blood pressure measurement - current techniques, office vs ambulatory blood pressure measurement. *Adv Exp Med Biol.* (2017) 956:85–96. 10.1007/5584_2016_49 27417699

[5] Graves JW Nash C Burger K Bailey K Sheps SG. Clinical decision-making in hypertension using an automated (BpTRU) measurement device. *J Hum Hypertens.* (2003) 17:823–7. 10.1038/sj.jhh.1001626 14704726

[6] Myers MG Valdivieso M Kiss A. Optimum frequency of office blood pressure measurement using an automated sphygmomanometer. *Blood Press Monit.* (2008) 13:333–8. 10.1097/MBP.0b013e3283104247 19020423

[7] Ohte N Saeki T Miyabe H Sakata S Mukai S Hayano J Relationship between blood pressure obtained from the upper arm with a cuff-type sphygmomanometer and central blood pressure measured with a catheter-tipped micromanometer. *Heart Vessels.* (2007) 22:410–5. 10.1007/s00380-007-0998-5 18044000

[8] Maloberti A Vallerio P Triglione N Occhi L Panzeri F Bassi I Vascular aging and disease of the large vessels: role of inflammation. *High Blood Press Cardiovasc Prev.* (2019) 26:175–82. 10.1007/s40292-019-00318-4 31054064

[9] McEniery CM Cockcroft JR Roman MJ Franklin SS Wilkinson IB. Central blood pressure: current evidence and clinical importance. *Eur Heart J.* (2014) 35:1719–25. 10.1093/eurheartj/eht565 24459197 PMC4155427

[10] Roman MJ Devereux RB Kizer JR Lee ET Galloway JM Ali T Central pressure more strongly relates to vascular disease and outcome than does brachial pressure: the Strong Heart Study. *Hypertension.* (2007) 50:197–203. 10.1161/HYPERTENSIONAHA.107.089078 17485598

[11] Pini R Cavallini MC Palmieri V Marchionni N Di Bari M Devereux RB Central but not brachial blood pressure predicts cardiovascular events in an unselected geriatric population: the ICARe Dicomano Study. *J Am Coll Cardiol.* (2008) 51:2432–9. 10.1016/j.jacc.2008.03.031 18565402

[12] McEniery CM Yasmin, Hall IR Qasem A Wilkinson IB Cockcroft JR Normal vascular aging: differential effects on wave reflection and aortic pulse wave velocity: the Anglo-Cardiff Collaborative Trial (ACCT). *J Am Coll Cardiol.* (2005) 46:1753–60. 10.1016/j.jacc.2005.07.037 16256881

[13] Takazawa K Kobayashi H Shindo N Tanaka N Yamashina A. Relationship between radial and central arterial pulse wave and evaluation of central aortic pressure using the radial arterial pulse wave. *Hypertens Res.* (2007) 30:219–28. 10.1291/hypres.30.219 17510503

[14] Kim G Kim JH Moon KW Yoo KD Kim CM Moon D The relationships between the arterial stiffness index measured at the radial artery and left ventricular diastolic dysfunction in asymptomatic high risk patients without atherosclerotic cardiovascular disease. *Int Heart J.* (2016) 57:73–9. 10.1536/ihj.15-225 26742882

[15] Kim G Kim JH Moon KW Yoo KD Ihm SH Youn HJ The clinical usefulness of central hemodynamics to evaluate diastolic dysfunction in subjects without hypertension. *Clin Interv Aging.* (2014) 9:527–33. 10.2147/CIA.S58810 24729693 PMC3974697

[16] Lang RM Bierig M Devereux RB Flachskampf FA Foster E Pellikka PA Recommendations for chamber quantification: a report from the American Society of Echocardiography’s Guidelines and Standards Committee and the Chamber Quantification Writing Group, developed in conjunction with the European Association of Echocardiography, a branch of the European Society of Cardiology. *J Am Soc Echocardiogr.* (2005) 18:1440–63. 10.1016/j.echo.2005.10.005 16376782

[17] Lewington S Clarke R Qizilbash N Peto R Collins R Prospective Studies C. Age-specific relevance of usual blood pressure to vascular mortality: a meta-analysis of individual data for one million adults in 61 prospective studies. *Lancet.* (2002) 360:1903–13. 10.1016/S0140-6736(02)11911-8 12493255

[18] Franco OH Peeters A Bonneux L de Laet C. Blood pressure in adulthood and life expectancy with cardiovascular disease in men and women: life course analysis. *Hypertension.* (2005) 46:280–6. 10.1161/01.HYP.0000173433.67426.9b 15983235

[19] Myers MG Campbell NR. Unfounded concerns about the use of automated office blood pressure measurement in SPRINT. *J Am Soc Hypertens.* (2016) 10:903–5. 10.1016/j.jash.2016.10.003 27863819

[20] Hougardy JM Leeman M. [Management of high blood pressure in patients with chronic kidney disease: summary of recent guidelines]. *Rev Med Brux.* (2016) 37:409–18. 28525209

[21] Filipovsky J Seidlerova J Kratochvil Z Karnosova P Hronova M Mayer O Jr. Automated compared to manual office blood pressure and to home blood pressure in hypertensive patients. *Blood Press.* (2016) 25:228–34. 10.3109/08037051.2015.1134086 26852625

[22] Group SR Wright JT Jr. Williamson JD Whelton PK Snyder JK Sink KM A randomized trial of intensive versus standard blood-pressure control. *N Engl J Med.* (2015) 373:2103–16. 10.1056/NEJMoa1511939 26551272 PMC4689591

[23] Whelton PK Carey RM Aronow WS Casey DE Jr. Collins KJ Dennison Himmelfarb C 2017 ACC/AHA/AAPA/ABC/ACPM/AGS/APhA/ASH/ASPC/NMA/PCNA Guideline for the prevention, detection, evaluation, and management of high blood pressure in adults: executive summary: a report of the American College of Cardiology/American Heart Association Task Force on Clinical Practice Guidelines. *J Am Coll Cardiol.* (2018) 71:2199–269. 10.1161/HYP.0000000000000075 29146533

[24] Williams B Mancia G Spiering W Agabiti Rosei E Azizi M Burnier M 2018 ESC/ESH Guidelines for the management of arterial hypertension: the Task Force for the management of arterial hypertension of the European Society of Cardiology and the European Society of Hypertension: the Task Force for the management of arterial hypertension of the European Society of Cardiology and the European Society of Hypertension. *J Hypertens.* (2018) 36:1953–2041. 30234752 10.1097/HJH.0000000000001940

[25] Group AS Cushman WC Evans GW Byington RP Goff DC Jr. Grimm RH Jr. Effects of intensive blood-pressure control in type 2 diabetes mellitus. *N Engl J Med.* (2010) 362:1575–85. 10.1056/NEJMoa1001286 20228401 PMC4123215

[26] Williams B Lacy PS Thom SM Cruickshank K Stanton A Collier D Differential impact of blood pressure-lowering drugs on central aortic pressure and clinical outcomes: principal results of the Conduit Artery Function Evaluation (CAFE) study. *Circulation.* (2006) 113:1213–25. 10.1161/CIRCULATIONAHA.105.595496 16476843

[27] Safar ME Blacher J Pannier B Guerin AP Marchais SJ Guyonvarc’h PM Central pulse pressure and mortality in end-stage renal disease. *Hypertension.* (2002) 39:735–8. 10.1161/hy0202.098325 11897754

[28] Ueda H Hayashi T Tsumura K Yoshimaru K Nakayama Y Yoshikawa J. The timing of the reflected wave in the ascending aortic pressure predicts restenosis after coronary stent placement. *Hypertens Res.* (2004) 27:535–40. 10.1291/hypres.27.535 15492471

[29] London GM Blacher J Pannier B Guerin AP Marchais SJ Safar ME. Arterial wave reflections and survival in end-stage renal failure. *Hypertension.* (2001) 38:434–8. 10.1161/01.HYP.38.3.434 11566918

[30] Sohn IS Ihm SH Kim GH Park SM Hong BK Lee CH Real-world evidence on the strategy of olmesartan-based triple single-pill combination in Korean hypertensive patients: a prospective, multicenter, observational study (RESOLVE-PRO). *Clin Hypertens.* (2021) 27:21. 10.1186/s40885-021-00177-z 34719392 PMC8559412

[31] Laurent S Cockcroft J Van Bortel L Boutouyrie P Giannattasio C Hayoz D Expert consensus document on arterial stiffness: methodological issues and clinical applications. *Eur Heart J.* (2006) 27:2588–605. 10.1093/eurheartj/ehl254 17000623

[32] Pagliai G Dinu M Madarena MP Bonaccio M Iacoviello L Sofi F. Consumption of ultra-processed foods and health status: a systematic review and meta-analysis. *Br J Nutr.* (2021) 125:308–18. 10.1017/S0007114520002688 32792031 PMC7844609

[33] O’Rourke MF. Carotid artery tonometry: pros and cons. *Am J Hypertens.* (2016) 29:296–8. 26687920 10.1093/ajh/hpv194

[34] Karamanoglu M O’Rourke MF Avolio AP Kelly RP. An analysis of the relationship between central aortic and peripheral upper limb pressure waves in man. *Eur Heart J.* (1993) 14:160–7. 10.1093/eurheartj/14.2.160 8449191

[35] Weber T Wassertheurer S Schmidt-Trucksass A Rodilla E Ablasser C Jankowski P Relationship between 24-hour ambulatory central systolic blood pressure and left ventricular mass: a prospective multicenter study. *Hypertension.* (2017) 70:1157–64. 10.1161/HYPERTENSIONAHA.117.09917 29061725

[36] Weber T Protogerou AD Agharazii M Argyris A Aoun Bahous S Banegas JR Twenty-Four-Hour Central (Aortic) systolic blood pressure: reference values and dipping patterns in untreated individuals. *Hypertension.* (2022) 79:251–60. 10.1161/HYPERTENSIONAHA.121.17765 34775789 PMC8654125

